# Biopharmaceutical characteristics of autologous red blood cells ghosts containing cytokines and antibiotics

**DOI:** 10.5195/cajgh.2013.103

**Published:** 2014-01-24

**Authors:** Zhaxybay Zhumadilov, Kulzhan Berikkhanova, Zarina Shulgau, Alexander Gulyayev, Zanybek Bokebayev, Nadiyar Mussin, Talgat Nurgozhin

**Affiliations:** 1 Center for Life Sciences, Nazarbayev University, Astana, Kazakhstan; 2 Astana Medical University, Astana, Kazakhstan

**Keywords:** autologous red blood cells, cytokines, antibiotics

## Abstract

**Introduction:**

Transport systems based on autologous red blood cells for targeted drug delivery can be considered as a promising approach in the treatment of surgical infections. Experimental studies have revealed the feasibility of targeted drug delivery by encapsulation of cytokines and antibiotics into autologous erythrocyte ghosts.

**Purpose:**

To study biopharmaceutical characteristics of autologous erythrocyte ghosts containing cytokines and antibiotics (pharmacocytes).

**Material and methods:**

The erythrocyte pharmacocytes were prepared by the hypotonic hemolysis method, or the use of human red blood cells. The association and dissociation indicators of rifampicin and cytokine substances with the erythrocyte ghosts were conducted using standard methods.

**Results:**

We have defined the following extracellular concentrations to be optimal for deposition of drug substances into pharmacocytes: for rifampicin – 10 000 μ/ml, erythropoietin - 1000 IU/ml, TNF-a - 5000 IU/ml, IL-1-β - 5000 U/ml, IFN-γ - 10 000ME/ml, IL-2 - 50 000 IU/ml, angiogenin - 0.04 mg/ml. Two types of correlations of cytokines and pharmacocytes were identified. In this study, we found that 40–60 % of the erythropoietin, IFN- γ and angiogenin were bound to red blood cells ghosts, more than 10% of which were bound irreversibly. For TNF-a, IL-1-β and IL-2, the red blood cells ghosts were capable of binding and depositing within 10–20 % of the input extracellular concentration, and these bindings were almost completely reversible. The rifampicin was bound by red blood cells ghosts with 5 % efficiency and also completely reversibly.

**Conclusion:**

The study has shown the effectiveness of inclusion of the studied components, such as erythropoietin, IFN-γ and angiogenin into the red blood cells ghosts, with significant efficiency (40–60 %). It presents the potential of using this system in targeted delivery of cytokines and antibiotics for treatment of surgical infections, thus facilitating the reduction in toxicity and adverse systemic effects of drugs and improving the treatment results.

